# In Vitro Evidence of Differential Immunoregulatory Response between MDA-MB-231 and BT-474 Breast Cancer Cells Induced by Bone Marrow-Derived Mesenchymal Stromal Cells Conditioned Medium

**DOI:** 10.3390/cimb45010020

**Published:** 2022-12-30

**Authors:** Víctor M. Arenas-Luna, Juan J. Montesinos, Víctor A. Cortés-Morales, José R. Navarro-Betancourt, Janneth Peralta-Ildefonso, Bulmaro Cisneros, Salomón Hernández-Gutiérrez

**Affiliations:** 1 Molecular Biology Laboratory, School of Medicine, Panamerican University, Mexico City 03920, Mexico; 2 Department of Genetics and Molecular Biology, Center of Research and Advanced Studies (CINVESTAV-IPN), Mexico City 04740, Mexico; 3 Mesenchymal Stem Cells Laboratory, Oncology Research Unit, Oncology Hospital, National Medical Center, IMSS, Mexico City 06720, Mexico

**Keywords:** breast cancer, mesenchymal stromal cells, immunoregulation, cytokines, conditioned medium

## Abstract

Inside tumors, cancer cells display several mechanisms to create an immunosuppressive environment. On the other hand, by migration processes, mesenchymal stromal cells (MSCs) can be recruited by different cancer tumor types from tissues as distant as bone marrow and contribute to tumor pathogenesis. However, the impact of the immunoregulatory role of MSCs associated with the aggressiveness of breast cancer cells by soluble molecules has not been fully elucidated. Therefore, this in vitro work aimed to study the effect of the conditioned medium of human bone marrow-derived-MSCs (hBM-MSC-cm) on the immunoregulatory capability of MDA-MB-231 and BT-474 breast cancer cells. The hBM-MSC-cm on MDA-MB-231 cells induced the overexpression of *TGF-β*, *IDO*, and *IL-10* genes. Additionally, immunoregulation assays of mononuclear cells (MNCs) in co-culture with MDA-MB-231 and hBM-MSC-cm decreased lymphocyte proliferation, and increased proteins *IL-10*, *TGF-β*, and *IDO* while also reducing TNF levels, shooting the proportion of regulatory T cells. Conversely, the hBM-MSC-cm did not affect the immunomodulatory capacity of BT-474 cells. Thus, a differential immunoregulatory effect was observed between both representative breast cancer cell lines from different origins. Thus, understanding the immune response in a broader tumor context could help to design therapeutic strategies based on the aggressive behavior of tumor cells.

## 1. Introduction

Worldwide, breast cancer is the most common malignancy in women and is considered the second leading cause of cancer-related death in women over 25 years of age. In 2020, 2.3 million new cases were diagnosed, representing 24.5% of all cancer types affecting the female population [1].

Breast cancer is considered a multifactorial disease with great cellular heterogeneity; diverse types of cells within the tumor engage in highly complex molecular interactions, with wide-ranging clinical implications [2]. Breast cancer is classified into various subtypes according to histopathology and at the molecular level, considering the expression of cell surface markers such as the estrogen receptor (ER), progesterone receptor (PR), human epidermal growth factor receptor 2 (ERBB2), among others, whose expression help to predict possible treatment responses [3,4,5]. Luminal and basal subtypes are highly prevalent, and the basal subtype is commonly referred to as triple-negative; the absence of these receptors determines their classification [3,6].

The tumor microenvironment is a dynamic and complex system in which cellular and non-cellular elements interact to regulate cancer progression. These elements include a wide range of soluble factors, such as cytokines, growth factors, chemokines, and cellular elements, such as immune cells, fibroblasts, endothelial cells, and mesenchymal stromal cells (MSCs), among others.

In breast cancer, as in other types of cancers, a high proportion of MSCs present in the tumor is recruited through migration processes from various tissues, the most common of which is the bone marrow [7,8]. MSCs are attracted to the tumor microenvironment through a chemotactic gradient comprised of soluble factors that include growth factors (e.g., *TGF-β*, EGF, PDGF, SCF, HGF, IGF-1 y GF, βFGF, HIF1α y VEGF) and some chemokine ligands (e.g., CCL2, CCL5, CCL22 y SDF-1α) secreted by tumoral cells [8,9,10,11,12,13,14,15,16,17]. In such a context, MSCs can promote and facilitate tumor growth, angiogenesis, metastasis, and chemoresistance and can modulate the antitumoral immune response [18,19].

In vitro, MSCs have an inhibitory effect on the proliferation of T lymphocytes [20]. MSCs under inflammatory conditions promote the differentiation of T lymphocytes towards a regulatory immunophenotype (Treg). In addition, other studies indicate that MSCs induce a protective effect on breast cancer cells by Treg cell promotion [21], thereby inhibiting the function of immune cells such as NK cells and lymphocytes, among others, through the expression of immunosuppressive factors including *IL-4*, *IL-10*, and *TGF-β* [22], whose presence could be a determinant in tumor pathogenesis and generate an immunosuppressive environment [23,24].

On the other hand, it is well known that cancer cells display either by cell contact or via paracrine a number of mechanisms to evade the immune system, which include the secretion of soluble molecules such as cytokines and growth factors; modifications to cell surface membrane-molecules expression [25], an increase in resistance to cell death pathways or creating an immunosuppressive environment through the induction and expansion of immunosuppressive Treg lymphocytes, which create a microenvironment that blocks the antitumor immune response [26] and could be a determinant factor in tumor pathogenesis [23,24].

Due to the serious technical and methodological difficulties, few studies to date have comprehensively analyzed the variety of molecular interactions that occur among different cell types within tumors. Thus, the use of breast cancer cell lines as an in vitro study model is important to understand the heterogeneity that characterizes breast cancer at the molecular and cellular levels [27]. For this reason, the selection of molecular markers representing each cell line from different subtypes is a key factor in breast cancer studies. On the other hand, it is known that each subtype of breast cancer can acquire particular characteristics that can be determined by the tumor microenvironment [28,29]. Although, multiple studies have been undertaken with the aim of understanding tumor cell biology the immunomodulatory effect that breast cancer cells with different aggressiveness characteristics may exert via interaction with MSCs has not been fully studied. Therefore, as a first approach to show a possible differential immunoregulatory response between breast cancer cell lines, we decided to evaluate using in vitro assays, the effect of human bone marrow-derived MSCs conditioned medium (hBM-MSC-cm) on the immunoregulatory properties of two breast cancer cell lines: the breast adenocarcinoma cell line with high-growing and highly metastatic potential MDA-MB-231(basal origin) triple-negative PR^-^, ER^-^ and ERBB2^-^ profile and the ductal breast cancer cell line with slow-growing and the weakly invasive BT- 474 (luminal origin) triple positive PR^+^, ER^+^ and ERBB2^+^ profile [30]. Our results indicate that MDA-MB-231 cells in co-culture with MNCs (lymphocytes) have an intrinsic capacity to increase the proportion of immunosuppressive molecules and Treg cells, which is enhanced by the presence of the hBM-MSC-cm, showing major immunosuppressive activity in vitro relative to BT-474 cells.

## 2. Materials and Methods

### 2.1. Cell Cultures

The MSC were isolated from human bone marrow by aspiration from the iliac crest of three hematologically healthy adult donors, according to the procedure established in the Traumatology and Orthopedics Hospital of the Mexican Institute of Social Security (protocol 1411) and were characterized according to the criteria established by the Interna-tional Society of Cell Therapy (ISCT) [31]. Once obtained, each bone marrow sample was placed in 50 mL conical tubes (Corning, New York, NY, USA) containing 15 mL of Roswell Park Memorial Institute (RPMI) culture medium supplemented with 10% fetal bovine serum (FBS). The cells were washed with phosphate-buffered saline (PBS, Thermo Fisher, Waltham, MA, USA) and separated on a density gradient with Ficoll-Paque Plus at a density of 1.077 ± 0.001 g/mL (GE Healthcare Bio-Sciences AB, Uppsala, Sweden). The cells were centrifuged at 1200 rpm for 30 min, and the interface was washed with PBS containing 3% FBS and 1 mM EDTA. The mononuclear cell pellet was resuspended in Dulbecco’s Modified Eagle’s Medium (DMEM; low glucose) supplemented with 15% FBS, 10 µL/mL glutamine (200 mM), 100 U penicillin, and 100 µg streptomycin. The number and viability of nucleated cells were determined with Turck’s solution and trypan blue (Thermo Fisher, Waltham, MA, USA), respectively; 5 × 10^6^ mononuclear cells were seeded onto 100 mm Petri dishes (Corning, New York, NY, USA) and incubated at 37 °C with 5% CO_2_ and 90% humidity. After 4 days, the cells were washed with PBS, and the medium was replaced twice per week. When the cultures reached 80–90% confluence, the cells were harvested and seeded to be expanded. The experiments were carried out in passage ≤10.

All antibodies in this work were used according to the supplier’s instructions and are shown in Table 1.

### 2.2. Characterization of hBM-MSC

#### 2.2.1. Immunophenotype

hBM-MSC were characterized according to the criteria established by the ISCT [31]. Conjugated monoclonal antibodies against CD73, CD90, CD45, CD31, CD73, CD14, CD105, HLA-A and HLA-DR were used to analyze the cells by flow cytometry.

To analyze the morphology of the hBM-MSC, 0.3 × 10^6^ hBM-MSC/cm^2^ were seeded onto P35 Petri dishes at 40% of confluence (Corning, New York, NY, USA), after which the cells were fixed with 4% PFA, stained with toluidine blue (Sigma-Aldrich, St. Louis, MI, USA), and observed by microscopy Axiovert, Zeiss (Carl Zeiss AG, Oberkochen, Stuttgart, Germany).

#### 2.2.2. Differentiation to Adipocytes

To evaluate the differentiation potential of hBM-MSC, 0.8 × 10^5^ hBM-MSC were seeded in low glucose DMEM medium (Thermo Fisher, Waltham, MA, USA) supplemented with 10% FBS in P35 Petri dishes (Corning, New York, NY, USA). Upon reaching 60% confluence, the cells were induced with MesenCult Adipogenic Differentiation Kit medium (STEMCELL Technologies, Vancouver, Canada) and incubated for 21 days. The medium was replaced twice per week. To visualize lipid vacuoles, cells were stained with Oil Red O solution (Sigma-Aldrich, St. Louis, MI, USA).

#### 2.2.3. Differentiation to Chondrocytes

3 × 10^5^ hBM-MSC were placed in 15-mL tubes and resuspended in low-glucose DMEM medium (Gibco, Thermo Fisher Scientific, Waltham, MA, USA) supplemented with 10% FBS. The tubes were centrifuged at 300× g to obtain a cellular pellet. The supernatant was removed and chondrogenic differentiation medium supplemented with TGF-β (Cambrex Bio Science, Walkersville, MD, USA) was added. The pellet was incubated for 28 days. The micromass was fixed, hydrated, and embedded in Tissue-Tek (Sakura CA, USA), after which 4-µm slices were cryosectioned and stained with Alcian Blue (Sigma-Aldrich, St. Louis, MI, USA) to evaluate the presence of chondroitin sulfate.

#### 2.2.4. Differentiation to Osteocytes

0.8 × 10^5^ hBM-MSC were seeded in P35 Petri dishes (Corning, New York, NY, USA) with low-glucose DMEM medium (Thermo Fisher, Waltham, MA, USA) supplemented with 10% FBS at 60% confluence. The cells were incubated with StemPro osteogenic medium (Gibco, Carlsbad, CA, USA) for 21 days. The differentiation medium was replaced twice per week. Osteocyte analysis was performed with Alizarin Red staining, which detects calcium deposits (Sigma-Aldrich, St. Louis, MI, USA).

### 2.3. Conditioned Media Preparation

To obtain the conditioned medium, hBM-MSC were grown until they reached 70–80% confluence, after which the media were replaced with FBS-free media. After 48 h, the medium was collected and centrifuged for 5 min at 2000 rpm and filtered with a 0.22-μm membrane (Millex GS; Millipore, MA, USA). The conditioned medium was either used immediately or frozen at –80 °C until use. A 2:3 mixture of conditioned medium plus fresh medium was prepared for experimental use as Sepehr et al. previously reported [32].

### 2.4. Breast Cancer Cell Lines

The breast cancer cell lines MDA-MB-231 and BT-474 were purchased from ATCC and grown in RPMI 1640 medium (Biowest, Missouri, MO, USA) supplemented with 10% FBS, 2 mM L-glutamine, 25 mM Hepes, 100 U penicillin, and 100 μg streptomycin in a 5% CO_2_ atmosphere and 90% humidity at 37 °C. The experiments were carried out in passage ≤13.

### 2.5. qRT-PCR Assays

The breast cancer cell lines MDA-MB-231 and BT-474 were seeded onto 100 mm Petri dishes at 60–70% confluence. Subsequently, the medium was replaced with hBM-MSC-cm. After 48 h, the total RNA was extracted using TRIzol-chloroform and treated with DNAse I (Invitrogen, Carlsbad, CA, USA). The cDNA was synthesized from 1 µg of total RNA using an oligo (dT18), following the supplier’s instructions (RevertAid First Strand cDNA Synthesis Kit, Gibco, Thermo Fisher Scientific, Waltham, MA, USA). The relative expression levels of various genes were analyzed by qRT-PCR using the Maxima SYBR Green/ROX qPCR Master Mix Kit (Thermo Fisher Scientific, Waltham, MA, USA), and the expression of glyceraldehyde 3-phosphate dehydrogenase (GAPDH) was used as a normalization control. The amplification conditions were as follows: denaturation at 95 °C for 10 min, followed by 40 cycles of 95 °C for 15 s, 60 °C for 30 s, and 72 °C for 30 s. Finally, the relative expression was calculated using the 2^−ΔΔCT^ method [33]. The oligonucleotides for each analyzed gene were designed using the Oligo Analyzer Tool program and are shown in Table 2.

### 2.6. MNCs Proliferation Assays in Co-Cultures

To perform immunomodulation assays, peripheral blood-derived MNCs were obtained from healthy adult donors (*n* = 3). The peripheral blood was diluted with PBS (1:2), and the cells were separated in a density gradient with Ficoll-Paque Plus at a density of 1.077 ± 0.001 g/mL (GE Healthcare Bio-Sciences AB, Uppsala, Sweden). The cells were centrifuged at 1200 rpm for 30 min, after which the interface was collected and washed with PBS. Then, the MNCs were incubated for 2 h at 37 °C with 5% CO_2_ and 90% humidity to remove monocytes. The number and viability of nucleated cells were determined with Turck’s solution and Trypan Blue (Thermo Fisher, Waltham, MA, USA), respectively. The MNCs were maintained in RPMI 1640 medium supplemented with 10% FBS, 2 mM L-glutamine, 100 U penicillin, and 100 μg of streptomycin until use.

To evaluate the effect of hBM-MSC-cm on the immunomodulation of MDA-MB-231 and BT-474 cancer cells, lymphocyte proliferation assays were performed in 48-well plates over a period of 6 days. Previously, MNCs were stained with carboxyfluorescein succinimidyl ester (CFSE, Thermo Fisher, Waltham, MA, USA) and seeded in co-cultures with breast cancer cells. The ratios are 3:1 MNC/MDA-MB-231 and 2:1 MNC/BT-474 cells with or without hBM-MSC-cm growing in plates of 48 wells with a surface area of 0.95 cm^2^. Finally, MNCs were activated with phytohemagglutinin (7.5 μg/mL). The groups analyzed were: (1) activated MNCs (positive control); (2) MNCs plus tumor cells; (3) MNCs plus tumor cells plus hBM-MSC-cm and (4) MNCs plus hBM-MSC-cm. The co-culture cells were seeded in the following proportions: 1 × 10^5^ MNCs, 5 × 10^4^ BT-474 cells, and 3 × 10^4^ MDA-MB-231 cells.

Following the incubation period, the cells present in the co-cultures were prepared for the analysis of the proliferation of MNCs by flow cytometry. The cells were disaggregated and washed with PBS containing 1 mM EDTA and centrifuged at 1200 rpm for 5 min. Subsequently, the cells were blocked with FBS for 20 min at 4 °C and washed with PBS-EDTA. The cells were then incubated with CD3 antibody (lymphocytes T antigen) and 7AAD (exclude dead cells) in 100 µL of PBS-EDTA at 4 °C for 20 min. The proliferation of MNCs (lymphocytes CD3^+^) was analyzed on a FACSCanto II Flow Cytometer (BD Biosciences, San Diego, CA, USA), acquiring 10,000 events per sample. The cells obtained in the proliferation assays were normalized using activated MNCs as a proliferation control, whose value was considered 100%. The data were analyzed with the FlowJo™ v.10.7.1 program (Ashland, OR, USA).

### 2.7. Quantitative Analysis of Soluble Molecules 

The supernatants from each group were collected and frozen at –70 °C until use. The cytokine concentration was determined according to De la Rosa et al. using a Cytometric Bead Array kit (BD Biosciences, San Diego, CA, USA) [34]. A standard curve was developed by preparing serial dilutions for each analyzed molecule (0–500 pg/mL). Later, 10 μL of the beads that recognize the TNF, *IL-10*, and *IL-4* cytokines were added to 50 μL of each sample or standard sample. Then, the samples were incubated at room temperature and protected from light for 30 min. After three washes with 1 mL of the wash buffer, the samples were centrifuged at 1200 rpm, and the pellet was resuspended in 300 µL of the same buffer. The samples were analyzed in the FACSCanto II Flow Cytometer (BD Biosciences, San Diego, CA, USA), and the cytokine concentrations were determined with LegendPlex v7.1 software (San Diego, CA, USA).

The concentration of *TGF-β* was determined using the *TGF-β* Cytometric Bead Array Kit (BD Biosciences, San Diego, CA, USA) according to the supplier’s instructions. *TGF-β* serial dilutions were used to perform a standard curve (40–10,000 pg/mL). Subsequently, 17 μL each sample was activated with 3.4 μL HCl 1 N for 10 min at room temperature. Then, the samples were neutralized with 3.4 μL NaOH 1.2 N/Hepes 0.5 M. Next, 17 µL of activated samples or standard samples were incubated with capture beads for 2 h protected from light. At end of the incubation time, 300 μL of washing buffer was added and centrifuged at 1800 rpm for 5 min. Finally, the samples were incubated with PE detector reagent at room temperature for 2 h and a second wash was performed before being analyzed in the Spectral flow cytometer Aurora (Cytek Biosciences, Fremont, CA, USA), and *TGF-β* concentration was determined with LegendPlex v7.1 software (San Diego, CA, USA).

### 2.8. Detection of IDO Expression in Breast Cancer Cells

The same groups assayed above were used to quantify by flow cytometry the intracellular *IDO* expression in breast cancer cells. After 6 days of co-culture, the cells were treated with Golgi Stop (BD Biosciences, San Diego, CA, USA) for 5 h. The cells in co-culture were harvested and labeled with CD45 antibody for 30 min at 4 °C, after which they were fixed and permeabilized with Fixation/Permeabilization Buffer (Sigma-Aldrich, St. Louis, MI, USA) for 1 h. Finally, the cells were labeled with anti-IDO for 30 min at 4 °C. The CD45^-^IDO^+^ cells were analyzed in a FACSCanto II Flow Cytometer (BD Biosciences, San Diego, CA, USA), 10,000 events were acquired from each sample and analyzed with the FlowJo™ v.10.7.1 software (Ashland, OR, USA).

### 2.9. Detection of Treg Cells

The same groups assayed above were used for flow cytometry to assess the proportion of regulatory T lymphocytes that have a CD4^+^CD25^+^FoxP3^+^ immunophenotype. After 6 days of co-culture, MNCs were harvested and labeled with CD4 and CD25 antibodies for 30 min at 4 °C, after which they were fixed and permeabilized with Fixation/Permeabilization Buffer (Sigma-Aldrich, St. Louis, MI, USA) for 1 h. Finally, the cells were labeled with FoxP3 for 30 min at 4 °C. The triple-positive cells were analyzed in a FACSCanto II Flow Cytometer (BD Biosciences, San Diego, CA, USA), 10,000 events were acquired from each sample and analyzed with the FlowJo™ v.10.7.1 software (Ashland, OR, USA).

### 2.10. Statistical Analyses

Nonparametric statistical tests were performed for all assays. Bonferroni-corrected pairwise comparisons were made for relative gene expression analyses. On the other hand, the assays of the relative proliferation of MNCs and the quantification of soluble factors were analyzed by one-way ANOVA with Bonferroni-corrected pairwise comparisons. Data analysis was performed using the Prism 8 program (GraphPad Software, Inc., San Diego, CA, USA). A *p*-value less than 0.05 was considered statistically significant.

## 3. Results

### 3.1. Characterization of hBM-MSC

**Figure 1:**
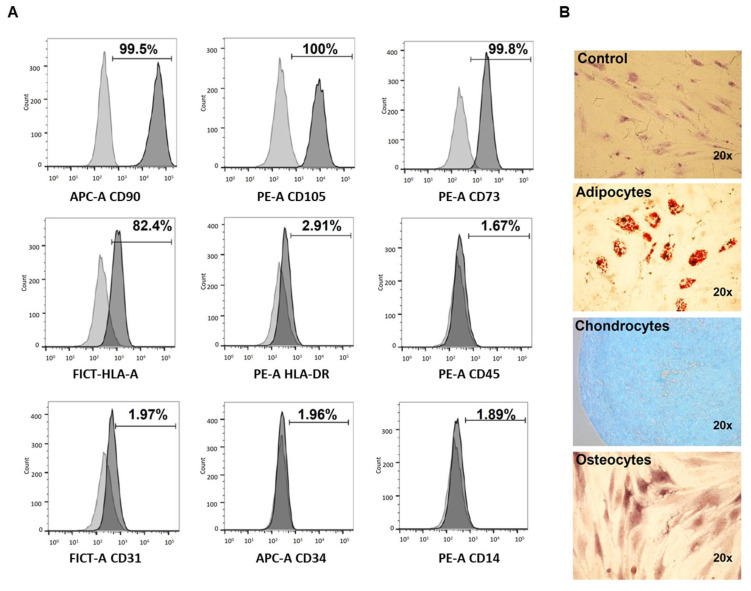
Characterization of human bone marrow mesenchymal stromal cell (hBM-MSC). (**A**) Representative histogram of immunophenotypes evaluated (*n* = 3) by flow cytometry, showing the surface markers evaluated. (**B**) Differentiation assay: hBM-MSC (control); adipocytes differentiated after 21 days, showing vacuoles stained with oily red; chondrocytes differentiated after 21 days, showing chondroitin sulfate staining with alician blue, and osteocytes differentiated after 28 days, showing calcium deposits stained by alizarin red.

Isolated primary cultures of hBM-MSC were characterized according to the criteria set forth by the ISCT [31]. The surface marker expression profile was analyzed by flow cytometry. The hBM-MSC showed a high expression of the cell-surface markers CD90, CD105, CD73, and HLA-A, whereas they did not express HLA-DR, CD45, CD34, CD14, and CD31 (Figure 1A). Afterward, their differentiation capability for the three basic lineages was evaluated, adding specific induction media (see Materials and Methods).

The hBM-MSC cultures showed adherent properties with fibroblast-like morphology (control), and they showed differentiation to the three basic lineages: adipocytes that were identified by the formation of lipid vacuoles; chondrocytes that evidenced the formation of the chondrogenic matrix and osteocytes by detecting the formation of calcium deposits (Figure 1B).

Once the identity of hBM-MSC was confirmed, we evaluated the influence of hBM-MSC-cm on the immunoregulatory properties of two breast cancer cell lines.

### 3.2. Effect of hBM-MSC-cm on the Expression of Immunomodulatory Genes in MDA-MB-231 and BT-474 Cells

**Figure 2:**
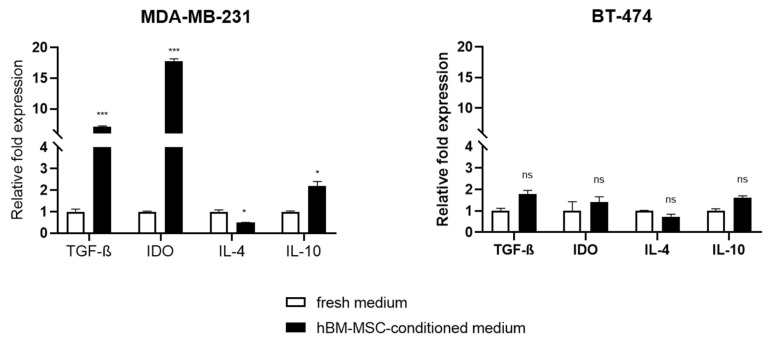
The hBM-MSC-cm induces the overexpression of immunomodulatory genes in MDA-MB-231 breast cancer cells. MDA-MB-231 and BT-474 cells were incubated with hBM-MSC-cm or fresh medium. After 48 h, RNA was isolated, and the expression level of *TGF-β*, *IDO*, *IL-4*, and *IL-10* was evaluated by qRT-PCR. The results are presented as the relative fold-change in expression. All values represent the average of three replicate from three independent experiments. Data are shown as mean ± SEM; * *p* < 0.05; *** *p* < 0.001.

It is known that cancer-derived cell lines produce a series of cytokines that can, in turn, modify the local immune response. Thus, to answer the question of whether hBM-MSC-cm can modulate the expression of some immunoregulatory genes, MDA-MB-231 and BT-474 cells were incubated with hBM-MSC-cm, and the expression levels of *TGF-β*, *IDO*, *IL-4*, and *IL-10* were evaluated by qRT-PCR (Figure 2). We observed a significant increase in the relative expression of the inflammatory genes *TGF-β* (7.1 ± 0.20), *IDO* (17.79 ± 0.61) and *IL-10* (2.19 ± 0.3) but not in *IL-4* (0.49 ± 1.02) in MDA-MB-231 cells incubated with hBM-MSC-cm compared to controls (fresh medium). In the case of BT-474 cells, no changes in the expression of the immunoregulatory genes were observed (Figure 2).

The modification of the expression levels of these genes induced by the hBM-MSC-cm suggests that MDA-MB-231 cells may possess a greater immunosuppressive capacity relative to BT-474 cells. Although both cell lines are derived from breast cancer, the effect exerted by the hBM-MSC-cm on each cancer cell line differed.

### 3.3. Proliferation Analysis of Mononuclear Cells Derived from Peripheral Blood as a Result in Co-Culture with Breast Cancer Cells Lines and hBM-MSC-cm

**Figure 3:**
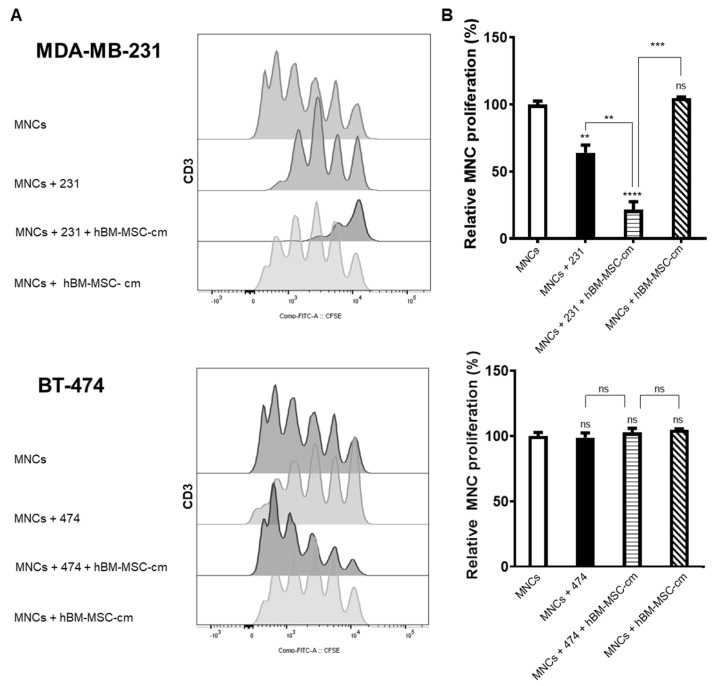
Immunomodulatory effect of breast cancer cells induced by hBM-MSC-cm. Mononuclear cells (MNCs) were stained with CFSE, activated with phytohemagglutinin, and co-cultured in the presence of MDA-MB-231 or BT-474, with or without hBM-MSC-cm for 6 days. The proliferation rate of activated MNCs (lymphocytes CD3^+^) was used as a positive control (100%). The figure shows (**A**) the histograms of a representative experiment, from three hBM-MSC-cm isolated from different donors (*n* = 3) (**B**) the graphs show the relative proliferation of MNCs (lymphocytes CD3^+^) from three independent experiments. Data are presented as mean ± SEM; ** *p* < 0.01; *** *p* < 0.001; **** *p* < 0.0001.

To analyze the effects of hBM-MSC-cm on the immunoregulatory capacity of tumor cells (MDA-MB-231 and BT-474), co-culture assays with MNCs-derived peripheral blood previously stained with CFSE and activated with phytohemagglutinin were performed. Then, the proliferation of MNCs (lymphocytes/CD3^+^) was evaluated. As is observed in Figure 3, in MDA-MB-231 cells, a marked decrease in the proliferation of MNCs was observed in two of the groups analyzed: (1) MNCs plus MDA-MB-23 cells and (2) MNCs plus MDA-MB-231 cells plus hBM-MSC-cm, even in the last group, we observed a strong decrease in the proliferation rate of MNCs. However, the hBM-MSC-cm did not show a proliferative effect on MNCs alone. Conversely, BT-474 cells did not affect MNCs proliferation in the groups analyzed, which attests to the inability of BT-474 cells to regulate the proliferation of lymphocytes under these conditions. As the expression assays denoted, the hBM-MSC-cm did not influence BT-474 cells to modify the proliferation of MNCs. In addition, no changes were observed in MNCs proliferation plus hBM-MSC-cm alone. Unlike BT-474 cells, MDA-MB-231 cells showed an immunoregulatory capability on MNCs proliferation, which is amplified when the hBM-MSC-cm was present.

### 3.4. Evaluation of Candidate Molecules Involved in the Regulation of MNCs Proliferation

**Figure 4:**
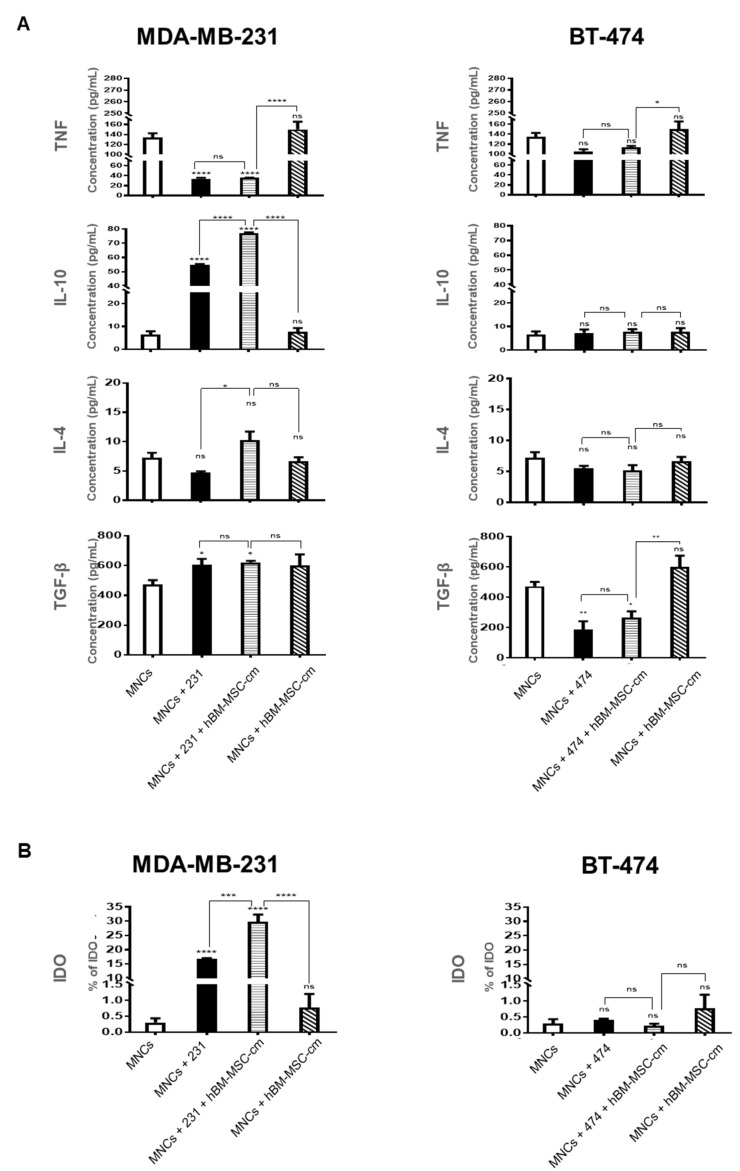
Quantification of cytokines and *IDO*. MNCs were activated with phytohemagglutinin and co-cultured for 6 days in the presence of MDA-MB-231 or BT-474, with or without hBM-MSC-cm (*n* = 3). (**A**) TNF, IL-4, *IL-10*, and *TGF-β* levels were quantified in the supernatants using cytometric bead array. The concentration of cytokines detected in the supernatant of activated MNCs (*n* = 3) was considered a basal concentration. (**B**) *IDO* was quantified as intracellular percentage in MDA-MB-231 and BT-474 cells by flow cytometry from three independent experiments. Data are presented as mean ± SEM; * *p* < 0.05; ** *p* < 0.01; *** *p* < 0.001; **** *p* < 0.0001.

To determine which molecules may be involved in inhibiting MNCs proliferation, the supernatant of the same groups assayed in Figure 3 were collected, and TNF, *IL-4*, *IL-10* and *TGF-β* were quantified (Figure 4A).

In MDA-MB-231 cells, TNF levels decreased in the following groups: (1) MNCs plus MDA-MB-231 cells and (2) MNCs plus MDA-MB-231 cells plus hBM-MSC-cm. However, TNF levels did not change significantly in the MNCs plus hBM-MSC-cm group. Contrary to TNF, the expression of *IL-10* increased in the following groups: (1) MNCs plus MDA-MB-231 cells and (2) MNCs plus MDA-MB-231 cells plus hBM-MSC-cm, with a substantial increase in the latter group. On the other hand, no significant changes in *IL-10* expression were found in the group with the presence of MNCs plus hBM-MSC-cm compared to the control. In addition, no significant changes in *IL-4* expression levels were found in any of the groups.

In MDA-MB-231 cells, the *TGF-β* levels were significantly increased in the groups: (1) MNCs plus MDA-MB-231 cells; (2) MNCs plus MDA-MB-231 cells plus hBM-MSC-cm and (3) the MNCs plus hBM-MSC-cm. However, no differences were observed among these three groups. The lower rate of MNCs proliferation in the presence of MDA-MB-231 cells could be explained by the decreased levels of the pro-inflammatory cytokine TNF and increased levels of the anti-inflammatory cytokine *IL-10* and *TGF-β*.

With respect to experiments using BT-474 cells, TNF levels did not change significantly in the following groups: (1) MNCs plus BT-474 cells and (2) MNCs plus BT-474 cells plus hBM-MSC-cm and (3) MNCs plus hBM-MSC-cm. Similarly, Figure 4A shows the concentration levels of *IL-10* and *IL-4*. Although the concentration levels of these cytokines differ, this pair of molecules present a similar tendency. However, when each group was analyzed, no statistically significant changes were found.

*TGF-β* analysis showed a significant decrease in the groups: (1) MNCs plus BT-474 cells and (2) MNCs plus BT-474 cells plus hBM-MSC-cm, but without difference between these two groups. On the other hand, no statistically significant difference was found in MNCs plus hBM-MSC-cm group in comparison with the control group.

### 3.5. hBM-MSC-cm Differentially Induces IDO Expression on MDA-MB-231 Breast Cancer Cell Line

To evaluate the effect of hBM-MSC-cm on the ability of cancer cells (MDA-MB-231 and BT-474) to produce immunosuppressive molecules in co-culture with MNCs, we quantified the expression of *IDO* intracellular in tumor cells by Flow cytometry (Figure 4B). The *IDO* protein levels were increased in MDA-MB-231 cells in the groups: (1) MNCs plus MDA-MB-231 cells, and (2) MNCs plus MDA-MB-231 cells plus hBM-MSC-cm. However, it is very striking that this last group has a higher expression of *IDO*. This fact confirms that the hBM-MSC-cm can exert a synergistic effect on the immunoregulatory properties of MDA-MB-231 cells possibly by significantly increasing *IDO* levels. In contrast, none of the co-culture groups analyzed with BT-474 cells showed significant changes in *IDO* protein expression levels.

### 3.6. Evaluation of the Effect of hBM-MSC-cm on the Capability of MDA-MB-231 and BT-474 Breast Cancer Cells to Generate Regulatory T Lymphocytes (CD4^+^CD25^+^FoxP3^+^)

**Figure 5:**
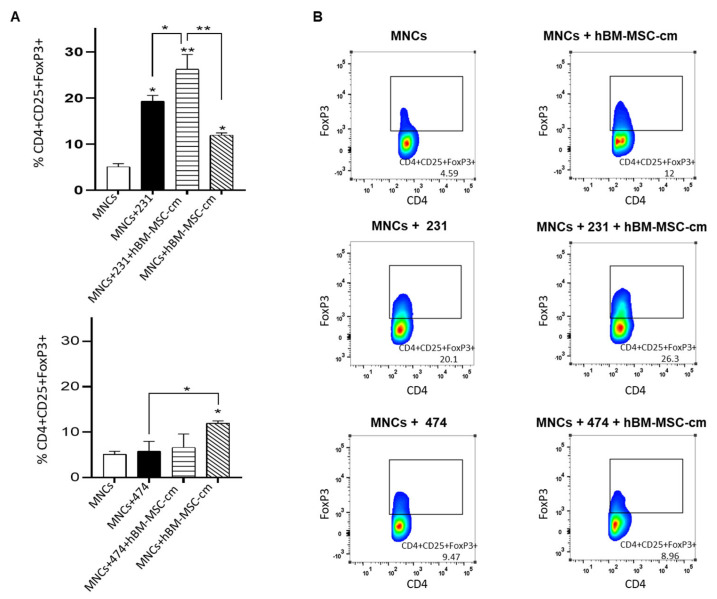
Capability of MDA-MB-231 and BT-474 breast cancer cells to generate regulatory T lymphocytes (CD4^+^CD25^+^FoxP3^+^) under the influence of hBM-MSC-cm (*n* = 3). (**A**) Once MNCs activated with phytohemagglutinin and co-cultured in the presence of MDA-MB-231 or BT-474, with or without hBM-MSC-cm. The average percentage of CD4^+^CD25^+^Foxp3^+^ cells was determined by flow cytometry after 6 days of culture. The population of CD4^+^CD25^+^Foxp3^+^ observed in the activated MNCs culture was considered as the control. (**B**) Plots representative of each group. The data show the mean ± SEM of the percentage of CD4^+^CD25^+^Foxp3^+^ cells from three independent experiments; * *p* < 0.05; ** *p* < 0.01.

The influence of MSCs throughout tumor development has been associated with the establishment of an immunotolerogenic environment via the release of soluble molecules. In this sense, MSCs can promote immune cell differentiation to certain regulatory phenotypes, whose presence can facilitate tumor development, inhibiting the antitumor immune response. The gene expression profile and cytokine concentration previously described in Figure 2 and Figure 4 showed that the immunomodulatory ability in both breast cancer cell lines could differ under the influence of hBM-MSC-cm. Therefore, we wondered whether these immunomodulatory mechanisms could involve the presence of regulatory T cells with a CD4^+^CD25^+^FoxP3^+^ phenotype (Figure 5). For BT-474 cells, no significant changes were found in the proportion of Treg cells in the following groups: (1) MNCs plus BT-474 cells and (2) MNCs plus BT-474 cells and hBM-MSC-cm. However, in the MNCs plus hBM-MSC-cm group, we observed an increase in the proportion of Treg cells, as expected, due to the presence of soluble factors which may be secreted by MSCs.

On the other hand, we observed a significantly higher proportion of Treg cells in the groups in presence of tumor cells MDA-MB-231 than those generated only in the presence of hBM-MSC-cm alone (Figure 5). However, the interaction between the hBM-MSC-cm plus MDA-MB-231 cells resulted in a significantly higher proportion of Treg cells, which did not occur with BT-474 cells. This result appears to corroborate the idea that hBM-MSC-cm could act synergistically and enhance the immunoregulatory power of MDA-MB-231 cells relative to that of BT-474 cells.

**Figure 6:**
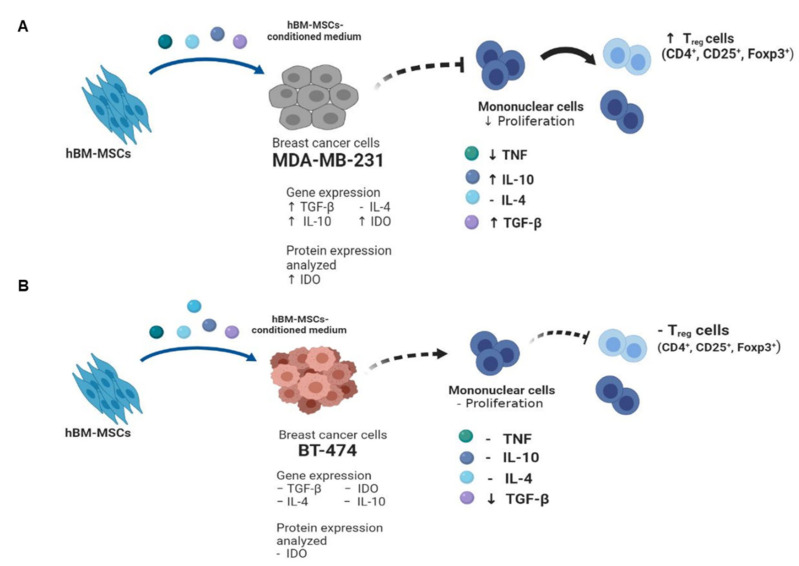
Proposed model of the differential effect of hBM-MSC-cm on the immunoregulatory ability of MDA-MB-231 and BT-474 breast cancer cells. The figure shows the possible interactions promoted by soluble factors secreted to the medium by hBM-MSC over (**A**) MDA-MB-231 and (**B**) BT-474 cells, affecting in a differential way the capability of each cell line to modulate the immune response. The MDA-MB-231 cells show a major suppressive potential relative to B-474 cells. (↑: increase; ↓: decrease; –: no modification). (Figure created with BioRender.com; December 26, 2022.

We propose a model base on our work to clarify the differential effect of hBM-MSC-cm on the immunoregulatory response of MDA-MB-231 and BT-474 breast cancer cells, via the secretion of soluble molecules (Figure 6). The breast cancer cell line triple-negative MDA-MB-231 exposed to hBM-MSC-cm overexpresses genes *TGF-β*, *IDO*, and *IL-10*. In immunoregulation assays of mononuclear cells (MNCs) in co-culture with MDA-MB-231 and hBM-MSC-cm, we see a reduction in lymphocyte proliferation along with increases in *IL-10* and *TGF-β* and reduced TNF levels; increases in intracellular *IDO* in MDA-MB-231 cells were also seen. Consequently, the presence of these molecules increases the proportion of regulatory T cells. In contrast, the hBM-MSC-cm does not affect the immunomodulatory capacity of luminal subtype BT-474 cells. Therefore, there is a differential immunoregulatory effect of MSCs on MDA-MB-231 and BT-474 breast cancer cells.

## 4. Discussion

In order to understand some of the possible effects of the cellular interactions that occur among different components of the tumor microenvironment, we analyzed the effect of hBM-MSC-cm on the immunomodulatory properties in vitro of two breast cancer cell lines: MDA-MB-231 and BT-474. These cell lines were carefully chosen for this work based on their clinical relevance and the subtype to which they belong. The BT-474 cells represent luminal-type breast cancer and are slow-growing. In contrast, the MDA-MB-231 cell line represents basal-type or triple-negative breast cancer, and is highly proliferative. Interestingly, triple-negative breast cancer patients have higher rates of metastasis, cell heterogeneity, and drug resistance. They have a worse prognosis and exhibit high morbidity and mortality rates [2]. Clinically, triple-negative cancer patients tend to have a recurrence within three years after primary surgery, while luminal breast cancer patients tend to have a recurrence a long time after primary surgery [35].

For this reason, as a first approach, we questioned whether through their conditioned medium, MSCs could differentially promote the expression of immunoregulatory genes such as *IDO*, *TGF-β*, *IL-4*, and *IL-10* in MDA-MB-231 and BT-474 cells lines, which may be crucial to favor the establishment of an immunotolerant state, because the overexpression of these immuno-regulatory genes correlate with a poor prognosis and, generally, are present in advanced tumor stages [36,37,38,39,40].

Our study found a marked overexpression of *IDO, TGF-β* and *IL-10* in MDA-MB-231 cells in the presence of hBM-MSC-cm, which could enhance the immunoregulatory capability due to the powerful suppressive effect of these gene products, as described in previous studies [41,42]. In fact, these molecules can induce the protection of tumor cells through the generation of Treg cells [43]. In BT-474 cells, low expression levels of these genes were found. In the case of *IDO*, its low expression in these cells has been related to the presence of estrogen receptors, which could induce hypermethylation of the *IDO* gene promoter [44].

The tumor microenvironment is characterized by the promotion of generalized dysfunction in T cells and, through some mechanisms can block their activation and function [45]. Therefore, we quantified the proliferation of MNCs (lymphocytes CD3^+^) as an effect established by the interaction that occurs between soluble factors present in the hBM-MSC-cm and both cancer cells. The results showed that BT-474 cells were unable to inhibit MNCs proliferation, whereas MDA-MB-231 cells had a powerful immunosuppressive effect, which may be associated with the observed expression of *TGF-β*, *IDO*, and *IL-10*.

Interestingly, it was observed that the hBM-MSC-cm added to the MDA-MB-231 cells (but not on BT-474) synergistically favored their immunomodulatory capability, since we found decreased levels of proliferating MNCs (lymphocytes CD3^+^) relative to those observed in the presence of MDA-MB-231 alone. Similarly, the proliferation values of MNCs using hBM-MSC-cm alone did not change significantly, possibly due to the lack of cell–cell contact (MNCs–MSCs), as previously reported [46,47].

Due to the decrease in the proportion of proliferating MNCs (lymphocytes CD3^+^) in some of the co-cultures with tumor cells plus hBM-MSC-cm, we decided to assess the levels of some cytokines present in the supernatants of the groups studied, because the balance between pro-inflammatory (e.g., TNF) and anti-inflammatory (e.g., *IL-4*, *IL-10*, and *TGF-β*) cytokines is key to controlling the regulation of the immune response [48]. This decrease in MNCs proliferation may be associated with the presence of Th2-type immunosuppressive cytokines, such as *IL-4*, *IL-10* and *TGF-β*, which are mainly responsible for immunosuppression in different types of cancer [49]. As our results showed, MDA-MB-231 cells have an intrinsic capacity to decrease the proliferation of MNCs, decrease TNF levels, and increase *IL-10*, and *TGF-β* levels and some of these effects were enhanced by hBM-MSC-cm presence. The result is relevant, given that the overexpression of IL-10 and *TGF-β* in cancer are key cytokines in the generation of an immunotolerant status, which affects the proliferation of T cells and avoids the maintenance of antitumor function [41,43,50,51]. On the other hand, in BT-474 cells, no evidence of a proliferative effect in MCNs was found; likewise, no significant changes were found in the concentrations of TNF, *IL-4*, and *IL-10* but curiously, we observed a decrease in *TGF-β* levels.

We also analyzed *IDO*-protein expression in tumor cells (MDA-MB-231 and BT-474), since *IDO* is considered a major contributor to tumor-induced immune suppression and a negative regulator of the immune system modulating T cell proliferation and immune tolerance associated with regulatory FoxP3^+^ T-cells in breast cancer [52]. The result observed in our work is interesting because the overexpression of *IDO* has been associated with aggressive tumors such as triple negative. At the same time, *IDO* is related to the expression of other immune response genes such as *IL-10* [53].

As MSCs, tumor cells have the ability to promote the generation of Treg cells (CD4^+^CD25^+^Foxp3^+^) through mechanisms that may or may not involve cellular contact between T lymphocytes and tumor cells or MSCs [22,41,54,55]. For this reason, we evaluated Treg cell populations in all experimental groups, MDA-MB-231 cells were found to have an intrinsic capacity to increase the proportion of Treg cells, which is enhanced by the presence of the hBM-MSC-cm, this could indicate a major immunosuppressive power of MDA-MB-231 cells in a tumoral context, comparing BT-474 cells, whose Treg cells levels did not change significantly.

Some reports indicated that basal or triple-negative tumor subtypes are surrounded by an inflammatory environment [22] in which MSCs may co-exist [56]. However, identifying the cells responsible for the overexpression of inflammatory mediator molecules has proved challenging [57]. In this context, our results could suggest that immunosuppressive molecules are produced by MDA-MB-231, and are possibly assisted by MSCs via soluble molecules.

In the tumor microenvironment where MSC are educated, Sepehr et al. described the major immunomodulatory potential of the conditioned medium of MSCs derived from breast tumors, versus the conditioned medium of MSCs derived from normal breast adipose tissue; these differences depend on the pathological or physiological context from which they were isolated. Interestingly, the hBM-MSC-cm used in our work did not promote an inhibitory effect on proliferation lymphocyte levels, as Sepehr et al. previously reported [32]. Despite that the hBM-MSC used in our experiments were not educated in a tumor environment, we did observe that the soluble agents secreted by hBM-MSC influence the immunoregulatory properties of MDA-MB-231 cells. Thus, it is important to study the immunoregulatory mechanisms that MSCs can influence in cancer, especially MSCs that are recruited from bone marrow to the tumor microenvironment because malignant cells mainly metastasize into bone tissue in advanced breast cancer [2,58,59]. Although several studies have described the immunosuppressive properties of MSCs, their capability to secrete a cocktail of proteins that modulate the immune response in vitro may be questionable because this protein profile could be modified by the culture conditions or even by donor inherent factors [60]. Thus, the study of molecules secreted by the MSCs could be decisive in the immunomodulatory response of the tumor microenvironment and remains an enormous challenge.

Based on the results obtained in this work, we propose a model that suggests a differential effect in the immunoregulatory properties of MDA-MB-231 and BT-474 breast cancer cell lines, interacting with hBM-MSC through soluble factors present in their conditioned medium.

However, it is necessary that future studies consider the other immunoregulatory mechanisms that different breast cancer cells may have under the influence of normal-MSCs and tumor-MSCs, either by paracrine signaling or cellular contact and determine whether the participation of MSCs inside the tumor microenvironment is key to the aggressive behavior in breast cancer. Likewise, it is necessary to elucidate in detail, the signaling pathways involved in the immunoregulatory effects observed.

In conclusion, our results demonstrated that hBM-MSC-cm may induce a differential effect on the immunoregulatory properties of MDA-MB-231 cells, but not BT-474 cells. Finally, understanding the immune response in the tumor context using a broader panel of tumor cell lines and clinical samples could help to correlate the breast cancer subtype with a possible immune response, which would be advantageous to design new therapeutic strategies.

## Figures and Tables

**Table 1:** Antibodies used for protein detection by flow cytometry.

Antigen	Company	Catalog Number
CD90 PE-Cy5	BD Biosciences, San Diego, CA, USA	555597
CD105 eFlour450	Ebioscience, San Diego, CA, USA	48-1057-42
CD73 PE-Cy7	BD Biosciences, San Diego, CA, USA	561258
HLA-A	Biolegend, San Diego, CA, USA	555552
HLA-DR PE-Cy-7	Biolegend, San Diego, CA, USA	307616
CD45 PE	BD Biosciences, San Diego, CA, USA	555483
CD31 FITC	BD Biosciences, San Diego, CA, USA	555445
CD34 APC	BD Biosciences, San Diego, CA, USA	555824
CD 14 PE	BD Biosciences, San Diego, CA, USA	555398
CD3 PE	BD Biosciences, San Diego, CA, USA	555333
7AAD	BD Biosciences, San Diego, CA, USA	559925
CD45 APC	BD Biosciences, San Diego, CA, USA	559865
IDO PE	RyD systems, Minneapolis, MN, USA	IC6030P
CD4 FITC	BD Biosciences San Diego, CA, USA	555346
CD25 PE	BD Biosciences San Diego, CA, USA	555432
FoxP3 PECy/7	Ebioscience, San Diego, CA, USA	25-4777-42

**Table 2:** Primers used for qRT-PCR gene expression.

Probe	Sequence	Amplicon Length (bp)
*GAPDH*	F FGGTGTGAACCATGAGAAGTATGA R GAGTCCTTCCACGA TACCAAAG	123
*IDO*	F AGGATTCTTCCTGGTCTCTCT R GTGTCCCGTTCTTGCATTTG	102
*TGF-β*	F CGTGGAGCTGTACCAGAAATAC R CACAACTCCGGTGACATCAA	112
*IL-10*	F GCTGGAGGACTTTAAGGGTTAC R GATGTCTGGGTCTTGGTTCTC	106
*IL-4*	F GTTCTACAGCCACCATGAGAA R CCGTTTCAGGAATCGGATCA	94

## Data Availability

Data supporting reported results can be found in the Molecular Biology Laboratory, Donatello 59 Cd de Mexico 03920, Panamerican University, Mexico.
